# Serum Amyloid Beta42 Is Not Eliminated by the Cirrhotic Liver: A Pilot Study

**DOI:** 10.3390/jcm10122669

**Published:** 2021-06-17

**Authors:** Reiner Wiest, Thomas S. Weiss, Lusine Danielyan, Christa Buechler

**Affiliations:** 1 Department of Visceral Surgery and Medicine, University Inselspital, 3010 Bern, Switzerland; reiner.wiest@insel.ch; 2 Children’s University Hospital (KUNO), Regensburg University Hospital, 93053 Regensburg, Germany; thomas.weiss@klinik.uni-regensburg.de; 3 Department of Clinical Pharmacology, Institute of Clinical and Experimental Pharmacology and Toxicology, University Hospital of Tuebingen, 72074 Tuebingen, Germany; lusine.danielyan@med.uni-tuebingen.de; 4 Department of Internal Medicine I, Regensburg University Hospital, 93053 Regensburg, Germany

**Keywords:** portal vein, MELD score, bilirubin, ascites, hepatic clearance, liver cirrhosis

## Abstract

Amyloid-beta (Aβ) deposition in the brain is the main pathological hallmark of Alzheimer disease. Peripheral clearance of Aβ may possibly also lower brain levels. Recent evidence suggested that hepatic clearance of Aβ42 is impaired in liver cirrhosis. To further test this hypothesis, serum Aβ42 was measured by ELISA in portal venous serum (PVS), systemic venous serum (SVS), and hepatic venous serum (HVS) of 20 patients with liver cirrhosis. Mean Aβ42 level was 24.7 ± 20.4 pg/mL in PVS, 21.2 ± 16.7 pg/mL in HVS, and 19.2 ± 11.7 pg/mL in SVS. Similar levels in the three blood compartments suggested that the cirrhotic liver does not clear Aβ42. Aβ42 was neither associated with the model of end-stage liver disease score nor the Child–Pugh score. Patients with abnormal creatinine or bilirubin levels or prolonged prothrombin time did not display higher Aβ42 levels. Patients with massive ascites and patients with large varices had serum Aβ42 levels similar to patients without these complications. Serum Aβ42 was negatively associated with connective tissue growth factor levels (r = −0.580, *p* = 0.007) and a protective role of Aβ42 in fibrogenesis was already described. Diabetic patients with liver cirrhosis had higher Aβ42 levels (*p* = 0.069 for PVS, *p* = 0.047 for HVS and *p* = 0.181 for SVS), which is in accordance with previous reports. Present analysis showed that the cirrhotic liver does not eliminate Aβ42. Further studies are needed to explore the association of liver cirrhosis, Aβ42 levels, and cognitive dysfunction.

## 1. Introduction

Amyloid-beta (Aβ) peptides of variable length are produced by proteolysis of amyloid precursor protein (APP). The two most common isoforms of Aβ are 40 and 42 amino acid peptides [1]. Cerebral accumulation of these Aβ peptides is a characteristic feature of Alzheimer disease [2]. Cell surface Low Density Lipoprotein Receptor-related Protein 1 (LRP1) and soluble LRP1 enhance the clearance of brain and circulating Aβ. In brain, LRP1 is involved in the transport of Aβ across the blood–brain barrier. LRP1 also mediates APP internalization and processing, and thereby contributes to Aβ generation [3]. LRP1 downregulation in brain microvessels of Alzheimer patients correlated with Aβ deposition in the brain [4]. To clarify the in vivo effect of LRP1 on Aβ production/clearance, mice expressing a mutant LRP1 protein, with impaired endocytosis and transcytosis activity, were crossed with a mouse model for Alzheimer disease (which were transgenic mice expressing mutant human APP with both the Swedish (K670N/M671L) and London (V717I) mutations) [3]. Mutant LRP1 finally led to reduced Aβ levels in cerebrospinal fluid and brain interstitial fluid and lower plaque burden [3]. This study provided evidence for a more prominent role of LRP1 in Aβ synthesis than degradation [3].

Recent evidence showed that the kidney, the skin, the gastrointestinal tract, and the liver contribute to peripheral Aβ clearance [5]. Aβ was detected in urine of humans, mice, and rabbits [6]. In a mouse model for Alzheimer disease (APP/Presenilin 1 mice expressing human mutant presenilin 1 and a chimeric mouse/human amyloid precursor protein (Mo/HuAPP695swe)) unilateral nephrectomy increased plasma and brain Aβ deposition and reduced Aβ in urine [6]. Whereas the molecules involved in renal clearance of Aβ have not been defined yet, hepatocytes express LRP1, which mediates Aβ uptake. Rat hepatocytes were shown to degradate these peptides or to excrete Aβ into the bile [7]. Detection of Aβ deposits in the human skin and intestine suggested that these tissues also contribute to peripheral clearance [8]. The exact pathways involved are still unknown [7]. Peripheral monocytes and tissue-resident macrophages eliminate Aβ peptides. Thus, tissue-resident macrophages may contribute to Aβ elimination not only in the skin and small intestine but also in tissues such as the liver [7]. Uptake and degradation of Aβ peptides by peripheral blood monocytes was, indeed, impaired in Alzheimer disease patients and this may also apply for tissue-resident phagocytes [9].

A separate study showed that Aβ levels in axillary lymph nodes of Alzheimer transgenic mice were as high as its brain concentrations and assumed that brain-derived Aβ40 and Aβ42 are cleared by lymphoid tissues. The mice used in this study expressed a human isoform of APP with the Swedish mutation, which causes high levels of Aβ and early onset of Alzheimer disease. Interestingly, Aβ40 and Aβ42 levels in the liver and kidney of these mice were hardly detectable [10].

Hepatic catabolism of Aβ42 was demonstrated in mice, and about 60% of the radiolabeled Aβ peptides accumulated in the liver [11]. Aβ42 protein levels were reduced in human and rodent cirrhotic liver tissues, and plasma Aβ42 levels were high in patients with liver cirrhosis. This may argue for a role of the liver in peptide clearing but may also mean that hepatic release of Aβ peptides is increased in cirrhosis [12,13]. Plasma Aβ42 positively correlated with bilirubin and aspartate aminotransferase (AST) levels and was negatively correlated with albumin, indicating an association between liver function and systemic levels of Aβ42 [12].

Impaired hepatic removal of blood Aβ42 can explain high plasma Aβ42 levels in patients with liver cirrhosis [12]. This may also contribute to low Aβ levels in the cirrhotic liver [12,13]. In the healthy human liver V-PLEX^®^ analysis detected 2.5–7.5 pg/mL Aβ42 levels, which declined about 10 fold in the cirrhotic liver [13]. Of note, hepatic Aβ protein was also low in Alzheimer disease. Aβ42 protein analyzed by ELISA in postmortem liver lysates of controls was 6.4–20.6 ng/g and was as low as 0–2.6 ng/g in the liver of patients with Alzheimer disease [14]. Studies on systemic Aβ42 levels in patients with Alzheimer disease have had mixed results [15]. Whether low hepatic levels are thus, indeed, linked to high systemic concentrations of Aβ42 has not been finally clarified [13,15].

It is important to note that Aβ42 exerts protective functions in the liver. Uptake of Aβ42 peptides by murine hepatic stellate cells suppressed the expression of fibrotic proteins such as transforming growth factor beta (TGFbeta) [13]. TGFbeta strongly contributes to tissue fibrosis and is an excellent inducer of connective tissue growth factor (CTGF) in hepatocytes. Serum CTGF is increased in patients with liver cirrhosis and is related to liver fibrosis [16,17,18].

Aβ further induced endothelial nitric oxide (NO) synthase in human SV40 immortalized hepatic sinusoidal endothelial cells [13]. NO is a vasodilatory molecule and its bioavailability is reduced in sinusoidal endothelial cells of the cirrhotic liver. This, indeed, contributes to increased intrahepatic vascular resistance and portal hypertension, which is one of the key factors responsible for the development of complications of liver cirrhosis such as varices and ascites [19,20,21]. Arginine and asymmetric dimethylarginine (ADMA) regulate endothelial function. ADMA is an endogenous inhibitor of NO synthase, whereas arginine can enhance production of NO [21,22]. Plasma levels of ADMA were higher in patients with decompensated than compensated liver cirrhosis, whereas arginine levels did not change [22,23].

There is some evidence that patients with chronic liver diseases have an increased risk for Alzheimer disease [24]. Non-alcoholic fatty liver disease (NAFLD) is a common disorder and may progress to liver cirrhosis and hepatocellular carcinoma [25]. NAFLD induced Alzheimer disease in wild-type mice and in mice carrying the Swedish APP protein and the Δe9 presenilin 1 mutation but lacking mouse APP protein [26]. Neuronal apoptosis, brain inflammation, and β-amyloid plaques were increased, whereas brain expression of LRP1 was reduced in both mouse groups when fed a high-fat diet to induce NAFLD [26]. Dyslipidemia and insulin resistance are characteristics of NAFLD, and abnormal lipid metabolism, as well as type 2 diabetes, increased the risk to develop Alzheimer disease [24]. A longitudinal cohort study identified higher brain Aβ levels in cognitively normal subjects who had abnormal triglyceride levels 20 years ago [27]. High low-density lipoprotein and low high-density lipoprotein levels were linked with higher cerebral Aβ in a cohort of patients with no or mild cognitive impairment [28]. Hypercholesterinemia, oxidative stress, and hyperinsulinemia impair Aβ clearance in type 2 diabetes patients, who have a higher risk of developing Alzheimer disease [7].

Analysis of protein levels in portal venous serum (PVS), hepatic venous serum (HVS), and systemic venous serum (SVS) gives some information about hepatic clearance and synthesis of different proteins and metabolites [29]. The liver can produce and eliminate metabolites and, thus, levels in peripheral blood might not represent the concentrations in portal or hepatic venous blood [29]. Different concentrations of metabolites in these blood compartments can also provide some information about their production in certain tissues. High synthesis of, e.g., cytokines in the gut or visceral adipose tissues may increase the concentration in the portal vein relative to the systemic blood [29,30]. In case that the production of the cytokine is induced in the liver, levels may be higher in the hepatic than the portal vein [29]. PVS and HVS are not available from healthy persons for ethical issues but can be collected from patients with liver cirrhosis during implantation of a transjugular intrahepatic portosystemic shunt. In these blood samples IL-6 levels were higher in PVS than HVS, demonstrating that IL-6 is cleared by the liver [31]. Impaired liver function was thus associated with higher levels of serum IL-6 [31]. IL-6 is an acute phase protein and regulates liver regeneration and metabolism. Permanently high IL-6 is, nevertheless, detrimental to liver health [32].

An increase of sCD163, which is almost exclusively produced by macrophages, from the portal to the hepatic vein showed that liver macrophages released this protein [33,34]. Plasma levels of sCD163 were positively related to the severity of liver disease in patients with liver cirrhosis and indicate activation of Kupffer cells in these patients [33,34].

Resistin in humans is also mainly released by macrophages, and serum levels were induced in liver cirrhosis. Resistin concentrations did, however, not differ between SVS, HVS, and PVS, suggesting that higher serum levels in liver cirrhosis are not a marker of Kupffer cell activation and may be related to the dysfunction of monocytes/macrophages in liver cirrhosis [35].

Chemerin is abundantly expressed in the liver and levels were higher in the hepatic than the portal vein [36,37,38]. Serum chemerin is a marker for hepatic dysfunction and was low in patients with liver cirrhosis [39,40].

Visfatin is an inflammatory and pro-fibrotic protein, and levels were higher in HVS and PVS when compared to SVS. Whether serum visfatin is changed in patients with liver cirrhosis or is related to liver disease severity has not been finally clarified [35].

Chronic inflammation contributes to the pathogenesis of Alzheimer disease and liver cirrhosis and, thereby, may link liver diseases with cognitive impairment [24,35]. C-reactive protein (CRP) is a routine laboratory marker for inflammation but was not increased in serum of patients with Alzheimer disease [41]. CRP is an acute phase protein synthesized by the liver. Patients with liver cirrhosis have higher systemic CRP. A relationship to the severity of the liver disease did not exist [42].

Here, HVS, PVS, and SVS of patients with liver cirrhosis were used to measure Aβ42. Only a little serum was available, and either Aβ40 or Aβ42 could be analyzed. Regarding that systemic Aβ42 is more strongly related to cognitive status [43], and that both peptides were comparably changed in plasma and liver of patients with liver cirrhosis [12,13], Aβ42 was determined.

It was hypothesized that serum Aβ42 is lower in HVS than PVS because of hepatic extraction. Clearance of Aβ42 by the liver may also contribute to lower serum levels. Moreover, it was postulated that Aβ42 levels increase in patients with more severe liver disease because of impaired removal by the injured liver.

## 2. Materials and Methods

### 2.1. Transjugular Intrahepatic Portosystemic Shunt (TIPS)

Twenty patients with clinically diagnosed liver cirrhosis were included in the study. Etiology of liver disease was alcoholic in 18 and hepatitis C infection in two patients. Patients were treated by TIPS implantation due to complications of liver cirrhosis. This procedure has been described earlier, and TIPS was inserted in the fasted state [44]. During this intervention, samples of the hepatic vein (HVS), of the portal vein (PVS), and of a peripheral vein (SVS) were obtained. Medication and alcohol consumption of the patients were not documented. Patients had not been drinking any alcohol at the time the blood samples were drawn.

Standard laboratory values (such as alanine aminotransferase (ALT), aspartate aminotransferase (AST), or albumin) were measured by the Institute for Clinical Chemistry and Laboratory Medicine at our hospital. The study complied with the Declaration of Helsinki. All patients gave written, informed consent and the study was approved by the ethical committee of the University Hospital of Regensburg.

G*Power3.1.6 analysis using the values of plasma Aβ42 in patients with cirrhosis (excluding patients with hepatitis B virus) and healthy controls published by Wang et al. [12] indicated that seven patients per group are enough to identify higher Aβ42 in patients with liver cirrhosis with an alpha error of 0.05 and a power of 0.80. More patients may be necessary to differentiate those with compensated and decompensated cirrhosis (because the difference in Aβ42 levels may be smaller in comparison to healthy controls) but there was no study having analyzed Aβ42 levels in serum of these patients so far and the number of patients needed could not be calculated.

### 2.2. ELISA

Human Amyloid β (aa1-42) Immunoassay was from R&D Systems (Wiesbaden, Nordenstadt, Germany). Serum was used undiluted. All of the blood samples were analyzed at one day in parallel. Therefore, inter-assay coefficient of variation (CV) could not be calculated. Intra-assay CV for samples with Aβ42 < 10 pg/mL was 12.4%, for samples with Aβ42 < 20 pg/mL was 12.3%, for samples with Aβ42 < 30 pg/mL was 8.1%, and for samples with Aβ42 < 100 pg/mL was 7.2%. The lowest value of the standard curve was 7.8 pg/mL, and almost all serum levels were greater. Median levels of Aβ42 were, indeed, about 20 pg/mL and were nearly three-fold higher than the lowest standard. Therefore, 50% of the analyzed sera had Aβ42 levels above the concentration of the second standard (15.6 pg/mL).

Interleukin (IL)-6, connective tissue growth factor (CTGF), chemerin, resistin, visfatin, arginine, and asymmetric dimethylarginine (ADMA) were already measured in patients with liver cirrhosis, and results were published [16,22,31,36,45].

Standards and samples were measured in duplicate, and the means were used for statistical analyses.

### 2.3. Statistics

Data are shown as box plots (median value, range of the values, the first and the third quartile—circles or stars outside the boxes indicate outliers). Shapiro–Wilk test showed that Aβ42 and chemerin were not normally distributed in the different blood compartments. Statistical differences were, therefore, calculated by non-parametric tests, namely the Mann–Whitney U Test, the Kruskal–Wallis Test, the Wilcoxon Test, or Friedman test (IBM SPSS Statistics 26.0). IL-6 was normally distributed, and paired *t*-test was used for calculation. Spearman correlations were analyzed by the IBM SPSS Statistics software.

## 3. Results

### 3.1. Aβ42 in Different Blood Compartments of Patients with Liver Cirrhosis

Serum of 20 patients suffering from clinically diagnosed liver cirrhosis was available for this study. Patients’ characteristics are given in Table 1.

**Figure 1:**
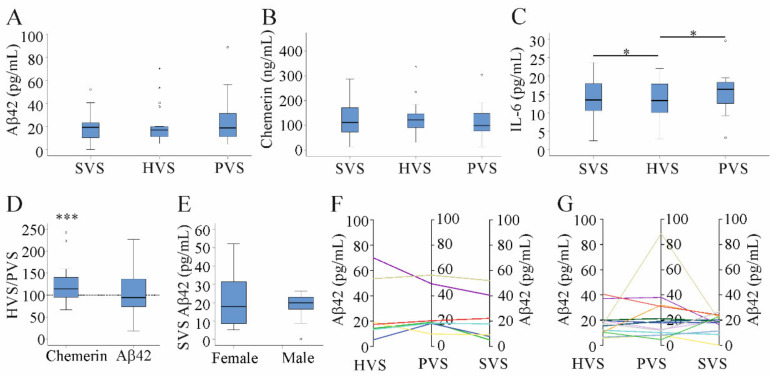
Aβ42, chemerin, and IL-6 in serum of patients with liver cirrhosis. (**A**) Aβ42, (**B**) chemerin, and (**C**) IL-6 in systemic venous (SVS), hepatic venous (HVS), and portal venous (PVS) serum of patients with liver cirrhosis. (**D**) HVS/PVS ratio of chemerin and Aβ42 in %. The dotted line is the 100% value. (**E**) SVS Aβ42 in females and males. (**F**) Aβ42 in HVS, PVS, and SVS of female and (**G**) male patients. * *p* < 0.05, *** *p* < 0.001.

Aβ42 levels were similar in SVS, HVS, and PVS (Friedman test; Figure 1A). A concern is that there were too few patients to identify any differences between the blood compartments. The number of patients was, indeed, too small to confirm higher levels of chemerin in HVS than PVS [36] (Friedman test; Figure 1B). In the current cohort, IL-6 was significantly higher in PVS than HVS, as was reported earlier. The recently described rise of IL-6 in SVS relative to HVS was also identified [31] (paired *t*-test; Figure 1C).

Small effects can be more easily identified when looking at the respective ratios. The mean of the chemerin HVS/PVS ratio was 123%, whereas the Aβ42 HVS/PVS ratio was 103%. The chemerin HVS/PVS ratio was increased relative to the value of 100%, which indicated that the ratio did not change (Wilcoxon Test; Figure 1D).

Aβ42 levels in SVS, HVS, and PVS did not differ between male and female patients (Mann–Whitney U Test; Figure 1E–G). Aβ42 levels in HVS, PVS, and SVS of the individual patients showed that levels of most patients were similar in the three blood compartments (Figure 1F,G).

It has to be noted that systemic Aβ42 levels did not correlate with patients’ ages (r = 0.312, *p* = 0.181). Median Aβ42 level in SVS of the two patients infected with hepatitis C virus was 16.1 pg/mL and was 19.7 pg/mL in the patients with alcoholic cirrhosis, suggesting that serum Aβ42 was not changed much by hepatitis C infection.

In general, there is a very good correlation between the metabolite concentrations in SVS, HVS, and PVS [29]. This was the case for chemerin and IL-6 levels in the current cohort (Table 2). Correlations for Aβ42 in SVS, PVS, and HVS were also significant. The correlation coefficients were, however, smaller (Spearman correlations; Table 2). A correlation coefficient of 0.838 for PVS and HVS chemerin corresponded to a coefficient of determination (R^2^) of 0.702, suggesting that about 70% of HVS chemerin can be explained by its PVS levels. R^2^ for PVS and HVS Aβ42 was 0.347, and about 65% of the variability in HVS must be related to other factors than the Aβ42 PVS levels [46].

### 3.2. Aβ42 in Relation to Scores and Measures of Liver Function

**Figure 2:**
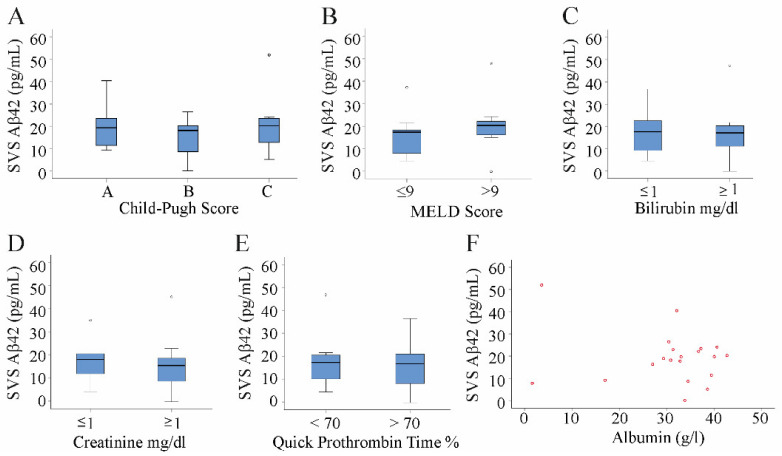
Aβ42 in relation to liver function. Aβ42 in serum of patients with liver cirrhosis stratified for (**A**) the Child–Pugh score (A = six patients, B = six patients, and C = eight patients) or (**B**) the median MELD score (nine patients had a MELD above 9 and 11 patients a MELD score ≤9). (**C**) Aβ42 serum levels in patients with a bilirubin value below (eight patients) or above (12 patients) the upper normal value (1 mg/dL). (**D**) Aβ42 serum levels in patients with creatinine levels below (eight patients) or above (12 patients) the upper normal value (1 mg/dL). (**E**) Aβ42 serum levels in patients with a normal (<70%, 10 patients) or a prolonged (>70%, 10 patients) Quick prothrombin time. (**F**) Spearman analysis showed that Aβ42 serum levels did not correlate with albumin in the 20 patients.

The calculation of the Child–Pugh score uses bilirubin, albumin, international normalized ratio (INR), ascites, and encephalopathy. The model for end-stage liver disease (MELD) score is calculated from bilirubin, INR, and creatinine [47]. Levels of Aβ42 were similar in patients with Child–Pugh scores A, B, and C (Kruskal–Wallis Test; Figure 2A). The median MELD score was 9, and Aβ42 did not differ between patients with a MELD score below or equal to 9 and patients with a MELD score above this median value (Mann–Whitney U Test; Figure 2B). Classification of patients according to normal and abnormal bilirubin levels, creatinine levels, or Quick prothrombin time did not reveal any differences in Aβ42 levels between the groups (Mann–Whitney U Test; Figure 2C–E). Accordingly, Aβ42 in serum did not correlate with serum albumin (Spearman correlation, Figure 2F).

### 3.3. Aβ42 in Patients with Disturbed Glucose Metabolism

**Figure 3:**
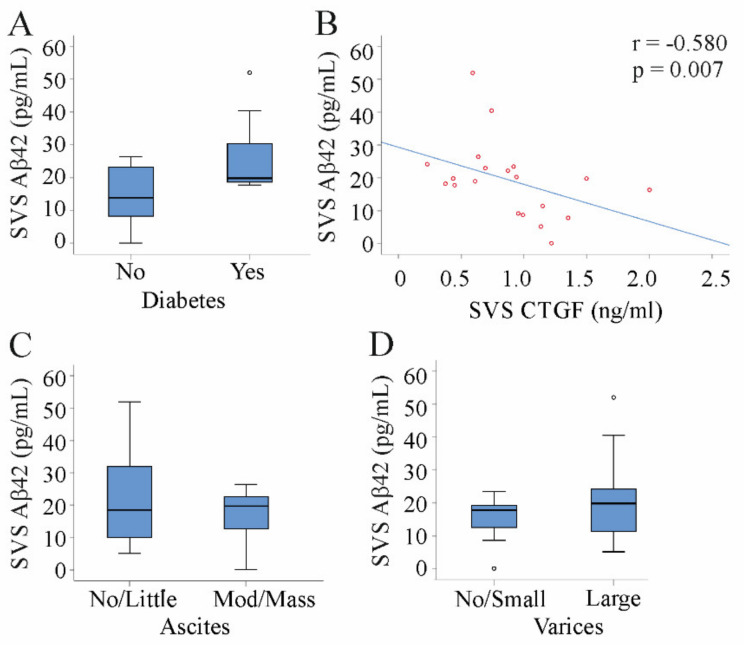
Association of Aβ42 serum levels with diabetes, ascites, and varices. (**A**) SVS Aβ42 serum levels in 8 patients with and 12 patients without diabetes. (**B**) Correlation of SVS Aβ42 with serum CTGF. (**C**) SVS Aβ42 serum levels in eight patients with no/little and 12 patients with moderate/massive ascites. (**D**) SVS Aβ42 serum levels in seven patients with no/small and 13 patients with large varices.

Type 2 diabetes increases the risk for Alzheimer disease [24,48]. In our cohort, diabetic patients had higher Aβ42 serum levels in comparison to the non-diabetic patients. This effect was significant in HVS (*p* = 0.047) but not in PVS (*p* = 0.069) and SVS (*p* = 0.181) (Mann–Whitney U Test; Figure 3A). HVS/PVS Aβ42 ratio (Mann–Whitney U Test; *p* = 0.910) did not differ between diabetic and non-diabetic patients.

### 3.4. Association of Aβ42 Levels with Markers of Inflammation, Endothelial Function, and Fibrosis

Systemic inflammation contributes to Alzheimer disease [49,50]. However, serum Aβ42 was not correlated with CRP (Table 3). Resistin, chemerin, IL-6, and visfatin were all described to be associated with inflammation [35,51,52,53], but serum Aβ42 did not correlate with any of these molecules (Spearman correlation; Table 3).

There was evidence that Aβ42 induced endothelial NO synthase production in the liver [13]. Serum arginine and ADMA are markers of endothelial function and were already measured in the serum of those patients [22,23,54]. Serum Aβ42 did not correlate with arginine and/or ADMA levels (Spearman correlation; Table 3).

Recent studies showed anti-fibrotic effects of Aβ42 [13]. CTGF was identified as a serum marker of ongoing fibrosis [17] and negatively correlated with SVS Aβ42 (Spearman correlation; Table 3, Figure 3B). HVS (r = −0.253, *p* = 0.283) or PVS (r = 0.038, *p* = 0.875) levels of these proteins were not correlated. Of note, HVS/PVS Aβ42 ratio negatively correlated with CTGF in PVS (r = −0.541, *p* = 0.014), HVS (r = −0.451, *p* = 0.046), and SVS (r = −0.489, *p* = 0.029).

### 3.5. Aβ42 in Patients with Ascites and Varices

Increased splanchnic and reduced hepatic NO production in liver cirrhosis contribute to portal hypertension [21]. Aβ42 did not correlate with the hepatic venous pressure gradient (Spearman correlation; r = 0.217, *p* = 0.358 for SVS; r =−0.77, *p* = 0.747 for HVS; r = −0.311, *p* = 0.182 for PVS). Esophageal varices and ascites are common complications of portal hypertension [55]. Systemic Aβ42 was similar in patients with modest/massive ascites in comparison to patients with no/little ascites (Mann–Whitney U Test; Figure 3C). Serum Aβ42 did also not change in patients with large varices in relation to patients with no/small varices (Mann–Whitney U Test; Figure 3D).

## 4. Discussion

This study showed that serum Aβ42 levels were similar in portal, hepatic, and systemic serum and were not related to measures of liver function in patients with liver cirrhosis.

Impaired elimination of Aβ has been proposed to contribute to elevated serum and brain levels, and thereby progresses in Alzheimer disease [24,56]. Recent studies suggested a role of the liver for Aβ degradation and removal [24]. Labeled Aβ42 peptides were, indeed, taken up by the liver and excreted in bile [11]. This finding gives, however, no information about the final levels of Aβ42 in the hepatic vein considering that the liver generates and degrades Aβ [13]. PVS and HVS Aβ42 levels were similar in the patients with liver cirrhosis. This suggests that the cirrhotic liver does not eliminate portal-venous Aβ42. It is, however, possible that Aβ42 is taken up by the cirrhotic liver for excretion but is also released from this organ into the circulation. APP mRNA and the enzymes for Aβ production are expressed in the liver [13]. Comparable Aβ42 levels in PVS and HVS are achieved when removal and production rates are similar. PVS and HVS levels of most cytokines and chemokines analyzed so far were highly correlated [16,22,31,36,45]. This also applied to chemerin and IL-6 levels in the small study cohort analyzed herein. Correlations of Aβ42 in SVS, PVS, and HVS were smaller and, thus, Aβ42 levels may be more extensively modified when passing the liver.

Whatever the underlying mechanisms are, the present findings indicated that levels of Aβ42 were not significantly reduced when passing the cirrhotic liver. Impaired liver function is associated with a diminished elimination of cytokines such as IL-6, and, accordingly, IL-6 levels increased with higher Child–Pugh score [31,57]. Aβ42 levels did not change in patients with worse liver function as assessed by the MELD score, the Child–Pugh score, and laboratory measures of liver disease severity. This illustrated that serum Aβ42 levels are not correlated with residual liver function in patients with cirrhosis.

It may well be that the healthy liver contributes to Aβ42 elimination. It is, however, difficult to obtain HVS and PVS blood from liver healthy donors. Wang et al. showed increased plasma Aβ42 levels in patients with liver cirrhosis in comparison to healthy controls [12]. This indeed suggests a function of the healthy liver in the regulation of plasma Aβ42 levels. The control group in that study was healthy subjects, and non-cirrhotic patients with chronic liver diseases were not included in the analysis by Wang et al. [12]. Patients infected with hepatitis B had about four-fold higher Aβ42 levels than non-infected patients. This shows that hepatitis B infection has a much greater effect on systemic Aβ42 levels than suffering from liver cirrhosis [12]. Current preliminary analysis suggested that hepatitis C infection (only two patients) was not associated with very high Aβ42 serum levels, but this needs further analysis.

It may well be that hepatic Aβ42 release is enhanced in patients with liver cirrhosis, and this may also reduce levels in the liver tissues and increase systemic concentrations of this peptide [12,13].

In an experimental murine NAFLD model, plasma Aβ42 even declined, and analysis of hepatic pathways involved in Aβ synthesis, catabolism, and clearance revealed that all of them were reduced in NAFLD [58]. Beta-secretase 1 was, however, found induced in the liver of db/db mice, which have a mutated leptin receptor and liver steatosis [59]. In human liver cirrhosis, beta-secretase 1 and neprilysin, which efficiently degrades the Aβ peptides, were suppressed, suggesting that production and degradation pathways were downregulated [13]. Chronic liver diseases may thus be associated with a dysregulation of Aβ production and removal. Major causes of liver cirrhosis are NAFLD, viral infection, and alcohol abuse [60]. Whether disease etiology may affect the hepatic production or clearance of Aβ42 was not studied so far. Data about expression of hepatic enzymes involved in Aβ42 metabolism and hepatic and systemic Aβ42 in patients stratified for etiology of liver cirrhosis are missing.

Aβ42 levels were about three fold higher in the cirrhosis patients studied by Wang et al. in comparison to our cohort. About 30% of their patients had chronic hepatitis B (HBV) [12]. Plasma Aβ42 levels in the non-HBV patients with liver cirrhosis were about 28 pg/mL, and this is comparable to the SVS Aβ42 levels (20 pg/mL) identified in our study where mostly patients with alcoholic cirrhosis were included.

The study by Wang et al. described associations of plasma Aβ42 with bilirubin, albumin, and AST concentrations [12]. The correlation analyses included data of non-HBV and HBV patients with liver cirrhosis and healthy controls, and analysis has to be done separately in these three different cohorts [12]. Positive associations of Aβ42 with markers of liver injury such as bilirubin and AST can also be explained by higher hepatic release of Aβ42 from the damaged liver, and a more detailed analysis is needed to characterize the pathways contributing to increased systemic Aβ42 in liver cirrhosis.

Higher age is a risk factor for liver cirrhosis and Alzheimer disease [2,35]. Aβ42 levels were, however, not correlated with age in the current cohort. A very modest negative association of plasma Aβ42 and age was described in cognitively normal subjects [61]. A second analysis showed a relatively weak positive correlation of plasma Aβ42 level and age [62]. These opposing reports suggest that correlations of Aβ42 level and age are weak and seem to be cohort specific.

Liver cirrhosis is often associated with chronic inflammation, and various cytokines were found induced in serum of these patients [35]. Plasma Aβ42 levels were, however, not correlated with IL-1β, IL-6, TNF, and IFN-γ in the study by Wang et al. [12]. In accordance with these findings, serum Aβ42 did not correlate with inflammatory proteins such as IL-6 and CRP in the current cohort. Therefore, it is unlikely that acute phase proteins or inflammatory proteins contribute to increased serum Aβ42.

There is evidence that Aβ42 is increased in obesity and diabetes. Obese mice had elevated plasma Aβ42 levels [63]. In diabetic patients, plasma Aβ42 levels were higher than in non-diabetic controls [63]. Of note, in the patients with liver cirrhosis studied herein, Aβ42 was significantly induced in HVS of diabetic patients with liver cirrhosis. HVS/PVS Aβ42 ratio did not differ between the diabetic and non-diabetic patients excluding that hepatic production was grossly induced in these patients.

Aβ42 was also shown to impair endothelium-dependent and -independent vasodilation and was associated with a lower NO bioavailability [63]. In the aorta of control and high-fat diet-fed mice, Aβ42 reduced phosphorylated endothelial NO synthase protein. On the other hand, it was shown that Aβ42 increased endothelial NO synthase protein in human liver sinusoidal endothelial cells [13]. It is well known that NO is a key factor in the hemodynamic abnormalities of liver cirrhosis. The reduced production of endothelial NO in the liver and its overproduction in the systemic and splanchnic vasculature are key factors for portal hypertension [21]. Common complications of portal hypertension are ascites and varices [19]. Aβ42 levels were not changed in patients with ascites or varices and did not correlate with the hepatic venous pressure gradient, serum arginine (NO precursor), or ADMA (NO synthase inhibitor) levels. Considering the complex regulation of NO synthesis and the multiple mediators that contribute to portal hypertension [21], this, however, does not exclude a role for Aβ42 in the regulation of splanchnic and hepatic NO production.

An interesting finding was the negative association of serum CTGF with Aβ42 levels. Serum CTGF was highest in patients with ongoing fibrogenesis [17]. Transforming growth factor β (TGF-β) is a strong inducer of hepatocyte CTGF synthesis [18]. Aβ42 reduces TGF-β in hepatic stellate cells [13], and this effect may contribute to the negative correlation of CTGF and Aβ42. HVS/PVS Aβ42 ratio was negatively correlated with CTGF in the three blood compartments, assuming a protective role of this peptide in liver cirrhosis.

The main limitation of this study is that only 20 patients were included. Thus, it was not possible to prove small differences. The use of an ELISA, instead of a highly sensitive technique such as the Single Molecule Array (Simoa)^®^ Aβ42 Advantage Kit, is a further limitation of this study. As mentioned above, Aβ42 levels detected in serum of our study cohort were comparable to the levels described in a previous analysis [12]. Moreover, the ELISA used herein was already applied for analysis of serum Aβ42 in a human study cohort [64]. Because of the low levels of Aβ42, serum had to be used undiluted and the specificity of the commercially available ELISA was not thoroughly checked. Medication and alcohol consumption of the patients were not documented and possible effects on serum Aβ42 could not be evaluated. The study strength is that portal vein, hepatic vein, and systemic blood of relatively well-characterized patients were used for analysis of Aβ42.

The present analysis showed that there is no net effect of the cirrhotic liver on Aβ42 levels in the circulation. Further studies are needed to explore the association of Aβ42 levels, ongoing fibrogenesis, and cognitive dysfunction in patients with liver cirrhosis.

## Figures and Tables

**Table 1:** Patient demographics and laboratory parameters (median values and ranges are shown).

	Cirrhosis Patients
Number	20
Sex (female/male)	7/13
MELD score	9 (6–21)
Age (years)	52 (40–81)
Child-Pugh score A/B/C/	6/6/8
C-reactive protein (mg/L)	9.9 (1.0–53.5)
Albumin (g/L)	32.9 (1.6–42.7)
Bilirubin (mg/dL)	1.2 (0.5–4.6)
Quick prothrombin time (%)	71 (28–100)
Aspartate aminotransferase (U/L)	40 (11–82)
Alanine aminotransferase (U/L)	33 (2–68)
Creatinine (mg/dL)	1.1 (0.5–4.5)
Ascites: no or little/modest or massive	8/12
Varices: no or small/large	7/13
Diabetes no/yes	12/8

MELD: Model for end-stage liver disease.

**Table 2:** Spearman correlation coefficients and *p*-values (in brackets) for chemerin, IL-6, and Aβ42 levels in the blood compartments.

	HVS Aβ42	PVS Aβ42
SVS Aβ42	0.541 (0.014)	0.463 (0.040)
HVS Aβ42		0.589 (0.006)
	HVS Chemerin	PVS Chemerin
SVS Chemerin	0.811 (<0.001)	0.912 (<0.001)
HVS Chemerin		0.838 (<0.001)
	HVS IL-6	PVS IL-6
SVS IL-6	0.950 (<0.001)	0.689 (0.001)
HVS IL-6		0.726 (<0.001)

HVS: Hepatic venous serum; IL-6: Interleukin-6; PVS: Portal venous serum; SVS: Systemic venous serum.

**Table 3:** Spearman correlation coefficients (r) and *p*-values (*p*) for the association of systemic Aβ42 with inflammatory markers, proteins with a role in nitric oxide production, and connective tissue growth factor (CTGF).

	CRP	Resistin	Chemerin	IL-6	Visfatin	Arginine	ADMA	CTGF
r	0.390	−0.011	−0.290	0.262	0.040	−0.251	−0.036	−0.580
*p*	0.089	0.965	0.214	0.531	0.867	0.286	0.880	0.007

ADMA: Asymmetric dimethylarginine; CTGF: Connective tissue growth factor; CRP: C-reactive protein; IL-6: Interleukin 6.

## Data Availability

The data presented in this study are available on request from the corresponding author.
